# Putative protective effects of sodium-glucose cotransporter 2 inhibitors on atrial fibrillation through risk factor modulation and off-target actions: potential mechanisms and future directions

**DOI:** 10.1186/s12933-022-01552-2

**Published:** 2022-06-28

**Authors:** Syona S Shetty, Andrew Krumerman

**Affiliations:** Montefiore Medical Center, 110 E 210th Street, Bronx, NY USA

**Keywords:** Sodium-glucose cotransporter 2 inhibitor, Atrial fibrillation, Epicardial adipose tissue, Left ventricular function

## Abstract

Atrial fibrillation, the most common cardiac arrhythmia, results in substantial morbidity and mortality related to its increased risks of stroke, heart failure, and impaired cognitive function. The incidence and prevalence of atrial fibrillation in the general population is rising, making atrial fibrillation treatment and management of its risk factors highly relevant clinical targets. One well-studied risk factor for the development of atrial fibrillation is diabetes mellitus. Inhibitors of sodium-glucose cotransporter 2 (SGLT2), common medications used to treat diabetes mellitus, have been observed to decrease the incidence of atrial fibrillation. This review discusses the SGLT2 and its role in glucose homeostasis, molecules inhibiting the transporter, possible physiological mechanisms responsible for the decreased incident atrial fibrillation in patients treated with SGLT2 inhibitors and proposes mechanistic studies to further our understanding of the biological processes involved.

## Introduction

Atrial fibrillation (AF) and atrial flutter (AFL) are the most common cardiac arrhythmias afflicting an estimated 59.7 million individuals worldwide in 2019 [1]. The Framingham Heart Study data indicates that AF-induced risk for stroke increases 4 to 5-fold and is associated with a doubling of mortality in both sexes [2]. The disease condition is also associated with a 3-fold risk of heart failure [3, 4] and a 2-fold risk of dementia and impaired cognitive function [5]. Despite concerted efforts to manage risk factors for AF [6–9], the prevalence and incidence of AF in the general population continue to rise [1].

Current pharmacotherapies targeting ion channels to treat AF have their own arrhythmogenic potentials [10, 11] and invasive interventional procedures (while highly effective), are not scalable given the vast numbers of AF patients needing treatment. Thus, a clear need exists for novel therapies or approaches addressing atrial fibrillation. A meta-analysis of published studies reaffirmed that patients with diabetes have an approximately 40% greater risk of AF compared to unaffected patients [12]. In this context, it is notable that dapagliflozin, a sodium-glucose cotransporter 2 (SGLT2) inhibitor approved for the treatment of patients with type 2 diabetes, decreased the incidence of AF/AFL in those patients [13].

In this review, we provide a brief background on SGLT2, its role in glucose homeostasis, molecules inhibiting the transporter, potential mechanistic basis for the apparent protective effects on AF by the inhibitors, and prospective studies to further elucidate responsible mechanisms.

### SGLT2 inhibition

The original finding that the glucose filtered and reabsorbed in human kidneys is sensitive to competitive inhibition by a naturally occurring compound called phlorizin [14] prompted research into the role of the kidneys in glucose homeostasis and diabetes. Preclinical studies in rats [15] validated the critical role of the kidney in glucose regulation and led to the identification of a low-affinity Na^+^-coupled glucose transporter in S1 segments and a high-affinity Na^+^-coupled glucose transporter located in S3 segments [16]. Kanai et al. [17] characterized the low-affinity, high-capacity Na^+^/glucose cotransporter that mediated saturable Na^+^-dependent and phlorizin-sensitive transport of D-glucose and labeled it SGLT2 to distinguish it from the high-affinity isoform SGLT1. SGLT2 expressed in the S1 segment of the nephron is responsible for approximately 90% of glucose reabsorption with the remainder done by SGLT1 in the S3 segment [18, 19]. The SGLTs are now known to comprise a family of active glucose transporter members [20] of which SGLT1 and SGLT2 are the most studied isoforms to date given their prominent role in glucose homeostasis and as logical drug targets for the treatment of diabetes.

Phlorizin, the prototypical SGLT1/SGLT2 dual inhibitor, while effective in lowering glucose levels in animal and human studies, is not an antidiabetic drug candidate given its poor pharmacodynamic and pharmacokinetic properties [21, 22]. Nonetheless, it served as a molecular starting point for the discovery and development of selective inhibitors of SGLT2 for the treatment of diabetes. This led to the synthesis of novel, relatively selective and efficacious SGLT2 inhibitors such as dapagliflozin, canagliflozin, empagliflozin, ertugliflozin, etc. [22, 23]. Canagliflozin was the first SGLT2 inhibitor approved by the US Food and Drug Administration (FDA) as an adjunct to diet and exercise to improve glycemic control in adults with type 2 diabetes mellitus [24–27]. This was followed by FDA approvals of dapagliflozin, empagliflozin, and ertugliflozin for the treatment of type 2 diabetes based on data from randomized clinical trials.

The FDA’s regulatory guidance of 2008 recommended outcome trials to rule out increased cardiovascular risk for all glucose-lowering therapies undergoing evaluation. Surprisingly, in the EMPA-REG OUTCOME trial, patients with type 2 diabetes at high risk for cardiovascular events who received empagliflozin, as compared to placebo, *had a lower rate* of the primary composite cardiovascular outcome and of death from any cause when the study drug was added to standard care [28]. This unexpected finding was supported by a meta-analysis of data extracted from six regulatory submissions and 57 published trials for seven SGLT2 inhibitors suggesting net protection by the SGLT2 inhibitor class of drugs against cardiovascular outcomes and death [29]. A multitude of randomized clinical trials [28, 30–40] has extended the range of disease conditions including heart failure and chronic kidney disease treatable with individual SGLT2 inhibitors. Furthermore, the SGLT2 inhibitor dapagliflozin was found to reduce the incidence of AF/AFL in patients with diabetes [13] or heart failure [41]. That the protective effects extended beyond dapagliflozin to include the broad class of SGLT2 inhibitors were indicated by most meta-analyses [42–47] with exceptions [48, 49]. Analysis of a large pharmacologic database also indicated a consistent and convincing reduction in reported AF with SGLT2 inhibition [50]. These results merit mechanistic studies and prospective, randomized clinical trials. Collectively, they may offer a better understanding of the pathophysiology of diabetes and reveal novel targets for exploration while adding to the therapeutic options for patients with AF. With this perspective, this review discusses plausible mechanisms that individually or in combination confer protection against AF by SGLT2 inhibitors.

### Mechanistic insights

SGLT2 is reportedly located almost exclusively in the epithelium of the renal proximal tubular segment with no detectable expression in the human heart [51–53]. Khemais-Benkhiat et al. [54] reported that SGLT2 expression is upregulated in cultured senescent endothelial cells of the porcine coronary artery when exposed to high glucose despite absence of expression under normal conditions, implying that the expression of the transporter could be induced under pathologic conditions in tissues with low basal expression. However, no expression of SGLT2 at both gene and protein level was found in tissue biopsies of healthy, ischemic, or hypertrophic human hearts [53]. Given these observations, the purported beneficial effect of SGLT2 on AF is apparently mediated by indirect metabolic or hemodynamic mechanisms triggered by its renal actions rather than by direct, ‘on-target’ effects on the cardiomyocyte. However, the possibility of ‘off-target’ actions of the different SGLT2 inhibitors directly on the atrial cardiomyocyte as a ‘class effect’ cannot be ruled out entirely given that they are structurally related as derivatives of phlorizin.

### Body weight reduction

The association of AF with obesity was recognized in the long-term prospective Framingham Heart Study [55]. A 4.7% linear increase in the risk of AF with each kg/m^2^ increase in body mass index (BMI) was evident in the Women’s Health Study [56]. Interestingly, women who were obese at baseline but then attained a BMI less than 30 kg/m^2^ by year 5 no longer had a significantly increased risk of subsequent AF in adjusted analyses in that study. This is substantiated by the observation in the LEGACY study that progressive weight loss has a dose-dependent effect on long-term freedom from AF in obese individuals with symptomatic AF [57]. In this context, it is interesting that therapy with SGLT2 inhibitors consistently results in a 1–3% body-weight loss in patients with type 2 diabetes [58] and that the effect is associated with a lower risk of new-onset AF [59]. The loss of about 1–3 kg body weight with treatment is characterized initially by a combination of early and rapid loss of water following osmotic diuresis associated with increased glucose excretion (calorie loss) and fat loss, and then by a maintained fat loss [58]. Dual-energy x-ray absorptiometry was used by Bolinder et al. [60] to demonstrate that the eventual weight loss in type 2 diabetes patients treated with dapagliflozin is the result of changes primarily in total-body fat mass, visceral adipose tissue, and subcutaneous adipose tissue. A study using bioimpedance spectroscopy confirmed the significant decrease in adipose tissue mass and fat tissue index without any observable change in lean tissue parameters in type 2 diabetes patients treated with dapagliflozin or empagliflozin [61].

The mechanistic basis for the association of obesity with AF is unclear. Obesity increases epicardial adipose tissue (EAT) mass overlying the posterior left atrium, atrioventricular groove, and infiltrating the atria [62–64]. Physiologically, the metabolically active fat depot is believed to release fatty acids through lipolysis for energy use by the heart under normal conditions–a process facilitated by a shared microcirculation given the lack of a muscle fascial plane between the fat depot and the myocardium [65, 66]. Under pathological conditions, dysfunctional EAT can release proinflammatory adipokines such as tumor necrosis factor alpha (TNF-α), monocyte chemoattractant protein-1 (MCP-1), interleukin-6 (IL-6), IL-1β, plasminogen activator inhibitor-1 (PAI-1), resistin, etc. [66–69], potentially contributing to fibrosis and structural and electrical remodeling of the impacted atrial myocardium [63, 70–73]. Interestingly, extracellular vesicles derived from epicardial fat specimens collected from patients with AF showed proinflammatory, profibrotic, and proarrhythmic signature suggesting that the vesicles may promote atrial fibrosis, the arrhythmogenic substrate for AF [74]. The Framingham Heart Study reported that pericardial fat (defined in the report as total adipose tissue within the pericardial sac; epicardial fat) but not other fat deposits was associated with prevalent AF even after adjustment for AF risk factors including body mass index [75]. The higher fat volumes were associated with approximately 40% higher odds of prevalent AF. Epicardial adipose tissue accumulation is associated with atrial pathophysiology predisposing to AF [76–80]. Dapagliflozin, the SGLT2 inhibitor reported to reduce the incidence of AF/AFL in patients with diabetes [13], also caused a substantial and rapid reduction in epicardial fat thickness [81]. Furthermore, treatment of patients with type 2 diabetes and coronary artery disease with dapagliflozin significantly decreased epicardial adipose tissue volume and P-wave indices such as P-wave dispersion and P-wave variation [82, 83]. A similar reduction in EAT volume was noted with empagliflozin following a sub-analysis of the EMPA-TROPISM trial in nondiabetic patients with heart failure with reduced ejection fraction [84]. The mechanism for the reduction in volume is unclear but is postulated to result from the glycosuric effects of the drugs, inducing a negative caloric balance and altering the ratio of glucagon and insulin to trigger enhanced lipolysis and reduction of epicardial adipose tissue [85]. Thus, SGLT2 inhibitor-mediated reduction in the volume of epicardial adipose tissue and potential detrimental remodeling effects on the atrial myocardium apparently contribute to the reported reduction in the incidence of AF, warranting prospective studies.

### Left ventricular function

Atrial fibrillation and congestive heart failure (HF) often coexist, with each condition predisposing to the other [86]. They share common risk factors including hypertension, diabetes, ischemic heart disease, obesity, and age. Atrial fibrillation occurs in more than half of individuals with HF and HF occurs in more than one third of individuals with AF at some point in time [87]. Amelioration of one often has favorable effects on the other. In the CASTLE-AF trial, AF ablation in heart failure patients led to a significant reduction of AF burden, an increase in left ventricular ejection fraction, and to a reduction in heart failure-related hospitalization and mortality [88]. Similar beneficial effects of AF-related ablation in patients with heart failure were noted in multiple studies [89–94]. However, the benefits of the procedure did not extend fully to patients with severely advanced heart failure [95]. Conversely, inhibitors of the renin-angiotensin-aldosterone system approved for the treatment of heart failure inhibit atrial remodeling and fibrosis and decrease incidence or recurrence of AF [96–99]. Thus, the observed benefits of SGLT2 inhibitors in reducing AF is seemingly secondary to the benefits of the drugs in cardiac function.

Empagliflozin treatment of individuals with diabetes and established cardiovascular disease produced a rapid and significant improvement in diastolic function [100]. A subsequent 3-month mechanistic study with the drug in that patient population revealed that the rapid, significant, and sustained improvement in left ventricular diastolic function as assessed by early mitral inflow velocity relative to early diastolic left ventricular relaxation (E/e’) was not accompanied by any change in systemic vascular resistance, cardiac index, stroke volume index, or pulse rate [101]. Similar improvements in left ventricular diastolic function were also observed within 3 months of treatment of patients with type 2 diabetes with canagliflozin [102], indicating that the improvement in diastolic function is a class effect of SGLT2 inhibitors. The apparent rapid improvement in left ventricular filling pressure implies that it is evoked by hemodynamic or metabolic alterations and not by structural changes in the tissue.

In patients with cardiac disease, tissue Doppler assessment of ventricular relaxation can be used to assess the effect of left ventricular relaxation in mitral E velocity, and the E/e’ ratio can be applied for the prediction of left ventricular filling pressures [103, 104] and atrial fibrillation [105]. As is well known from the work by Haissaguerre et al. [106], the cardiomyocytes in the pulmonary vein sleeves are a significant ectopic source of aberrant electrical signals triggering left atrial arrhythmogenesis. Increased left ventricular filling pressure exerts hemodynamic stress on the left atrium and pulmonary veins, especially during the ‘atrial kick,’ causing stretching of the resident cardiomyocytes. Stretch of the pulmonary veins triggers arrhythmogenesis in the left atrium [107, 108]. Significant dilation of both superior pulmonary veins and the left atrium reportedly occurs in patients with atrial fibrillation [109]. Accordingly, the incidence of AF is related to pulmonary vein volume index [110]. Thus, the structure and function of the left atrium are directly impacted by left ventricular filling pressure. Reduction of the hemodynamic stress on the left atrium and pulmonary veins by SGLT2 inhibitor-mediated improvement in left ventricular diastolic function may thus ameliorate the trigger mechanism and acutely inhibit AF.

The mechanism underlying the acute improvement in left ventricular function by SGLT2 inhibitors is unclear. The effect of empagliflozin is thought to result from osmotic diuresis with electrolyte-free water excretion leading to immediate cardiac preload reduction, suggested by reduced early mitral inflow velocity E [101]. A mathematical model-based investigation suggested that SGLT2 inhibitors achieve diuresis by a mechanism distinct from that of other diuretic classes [111]. SGLT2 inhibition apparently elicits greater fluid clearance from the interstitial fluid than from the circulation, potentially reducing elevated cardiac filling pressures without causing neurohumoral activation. Additionally, the increased availability of circulating ketone bodies from the glucagon-mediated ketogenesis [112], may lead to improvement in myocardial energetics [113]. Dapagliflozin reduced left ventricular mass in subjects with type 2 diabetes and left ventricular hypertrophy in a longer, 12-month study [114], indicating that it reverses pathologic cardiac remodeling to possibly improve diastolic function. A significant increase in hematocrit, thought to be from decreased plasma volume and resultant hemoconcentration, was also observed in that study–suggesting presence of an additional mechanism for enhancing oxygen supply to the tissue. There may be other mechanisms contributing to this as well. There is a large body of literature investigating the mechanism(s) potentially responsible for the beneficial effects of SGLT2 inhibitors in patients with heart failure with or without diabetes as summarized in several review articles [115–120] that could relate a reduction in AF via improvements in ventricular function. Finally, the AF-reducing effect is multifactorial with the early effects being triggered by acute improvement in diastolic function as described above and sustained later by reversal of adverse structural and electrical remodeling of the left ventricle, left atrium, and pulmonary veins.

### Antihyperglycemic effect and blood pressure lowering

The relationship between type 2 diabetes and new-onset AF is well documented [121]. A meta-analysis comprising 7 prospective studies and 4 case-control studies involving 1.7 million subjects indicated an approximately 40% higher risk of developing AF in patients with diabetes compared to unaffected patients. The increased risk remained significant at about 25% after adjustment for confounding multiple risk factors and publication bias [12]. The antihyperglycemic effects by the SGLT2 inhibitors were relatively modest [28, 31, 33, 34], suggesting that they are unlikely to account for the observed benefits of AF considering that intensive glycemic control *per se* does not affect the rate of new-onset AF [122].

Similarly, on a population level, elevated blood pressure is a risk factor for incident AF. A 20-mmHg increase in systolic blood pressure is associated with 21% higher risk of AF [123]. Intensive blood pressure lowering in patients with hypertension is associated with a 26% lower risk of developing incident AF [124]. A meta-analysis characterizing the blood pressure-lowering effects of SGLT2 inhibitors in 27 randomized clinical trials including canagliflozin, dapagliflozin, empagliflozin, ipragliflozin, and remogliflozin [125], revealed moderate reductions in both systolic blood pressure (approximately 5 mmHg) and diastolic blood pressure (approximately 2 mmHg) attributable to osmotic diuresis and natriuresis [126]. The magnitude of the reductions was essentially similar across the entire drug class. The dapagliflozin treatment-related reduced risk of AF was not modified by systolic blood pressure [13], suggesting that the marginally lowered blood pressure is unlikely to fully account for the beneficial effect.

### Possible ‘off-target’ actions on cardiomyocytes

As discussed earlier, SGLT2 is located almost exclusively in the epithelium of the renal proximal tubular segment with no detectable expression in the human heart [51–53]. However, studies using human, or animal cardiomyocytes have shown that empagliflozin inhibits the sodium-hydrogen exchanger (NHE) activity [127, 128] similar in magnitude to that produced by cariporide [127], a selective inhibitor of the exchanger [129]. Molecular binding studies have demonstrated high binding affinities of SGLT2 inhibitors possibly with the extracellular Na^+^-binding site of the NHE [130], suggesting that an ‘off-target’ effect of the inhibitors on cardiomyocyte ionic homeostasis may contribute to the beneficial effects. The NHE is a major regulator of intracellular pH under normal physiological conditions as well as during pathological conditions such as cardiac ischemia or cardiac hypertrophy/remodeling [131, 132]. It mediates extrusion of protons from the cell with a concomitant increase in intracellular Na^+^ concentration. This is expected to activate the reverse mode of the Na^+^/Ca^++^ exchanger to increase intracellular Ca^++^, cause Ca^++^ overload, induce atrial electrical remodeling, and predispose to AF. Inhibition of the NHE in such a situation would be expected to interrupt this sequence of events and inhibit AF [133]. However, results from experimental studies examining the effects of NHE inhibitors in animal models of AF have been mixed. Jayachandran et al. [134] reported that blockade of the cardiac NHE by HOE648 (cariporide) blocked the shortening of the atrial effective refractory period in dogs subjected to rapid atrial pacing, suggesting a beneficial effect of the agent against AF. In contrast, Shinagawa et al. [135] observed no benefit by cariporide against atrial remodeling in dogs exposed to seven days of rapid atrial pacing. Similarly, in a goat model of AF, the NHE inhibitors EMD87580 or EMD125021 did not prevent or revert AF-induced atrial remodeling, leading the investigators to conclude that blockers of the exchanger are unlikely to be of benefit in the prevention or treatment of AF [136]. Thus, the role of SGLT2-inhibitor-mediated block of the NHE in preventing AF is currently unclear, warranting further investigation.

Shao et al. [137] have postulated that empagliflozin is potentially useful in the prevention of diabetes-related AF as it can improve mitochondrial function and biogenesis, and prevent atrial structural and electrical remodeling in a high-fat diet/streptozotocin rat model of insulin resistance, obesity, and diabetes. There was expectedly a significant reduction in body weight, and blood glucose in the treated rats relative to the control diabetic animals in that study. Mitochondria in the diabetic human atrium do show impaired capacity to oxidize fatty acids and glutamate and indicate persistent oxidative stress [138]. Whether the improved mitochondrial function and biogenesis observed in the study by Shao et al. [137] are secondary to the beneficial metabolic changes produced by the known pharmacological actions of the drug or to direct effects on mitochondrial dynamics are yet to be ascertained.

### Future directions

The post hoc analysis of the DECLARE-TIMI 58 trial indicating decreased incidence of AF in high-risk patients with type 2 diabetes treated with dapagliflozin [13] is hypothesis-generating since it was not the prespecified primary outcome in the trial. Ad﻿equately powered, prospective randomized clinical trials with AF (new onset, progression, or reversal) as the primary outcome are needed to extend the findings. Determination of the timeline of new-onset AF in asymptomatic subjects even with established clinical risk factors is challenging without continuous rhythm monitoring. The value of this parameter is questionable as well, since it is often dependent on an initiating trigger which may occur by chance (indicating that AF burden instead may be a better approach) [139]. Examining the effect of SGLT2 inhibitors on the time-to-first recurrence of AF after ablation or cardioversion may be a more pragmatic approach given the increased likelihood of AF recurrence in this patient population.

SGLT2 inhibitors elicit pleotropic effects. It is critical to identify the principal driver of the drug-mediated reduction in incident AF. Delineation of the beneficial structural and functional changes in various organ systems relevant to AF pathogenesis as highlighted in this review will facilitate subsequent investigations of the molecular basis for the beneficial effects of the drugs on AF.

Comparative proteomic analysis of EAT samples biopsied before and after drug treatment may yield insights to specific effects on pathways related to inflammation, fibrosis, apoptosis, arrhythmogenesis, etc. Inflammatory cytokines such as TNF-α, IL-1, IL-6 directly modulate the function of cardiac ion channels to promote arrhythmia [140]. For instance, TNF induces atrial structural remodeling and downregulation of connexin40 to promote arrhythmia in a transgenic mouse model with cardiac targeted overexpression of TNF [141]. Valuable information may be obtained by investigating the relationship between changes in left ventricular relaxation (E/e’ ratio) and left atrial strain in diabetic patients with or without AF upon treatment with SGLT2 inhibitors. Left atrial strain measurements may provide early detection of functional changes before any changes in left atrial dilation can be detected [142]. Furthermore, left atrial strain or strain rate can predict postoperative AF, AF recurrence after ablation, and facilitate grading of diastolic dysfunction [143].

**Fig. 1:**
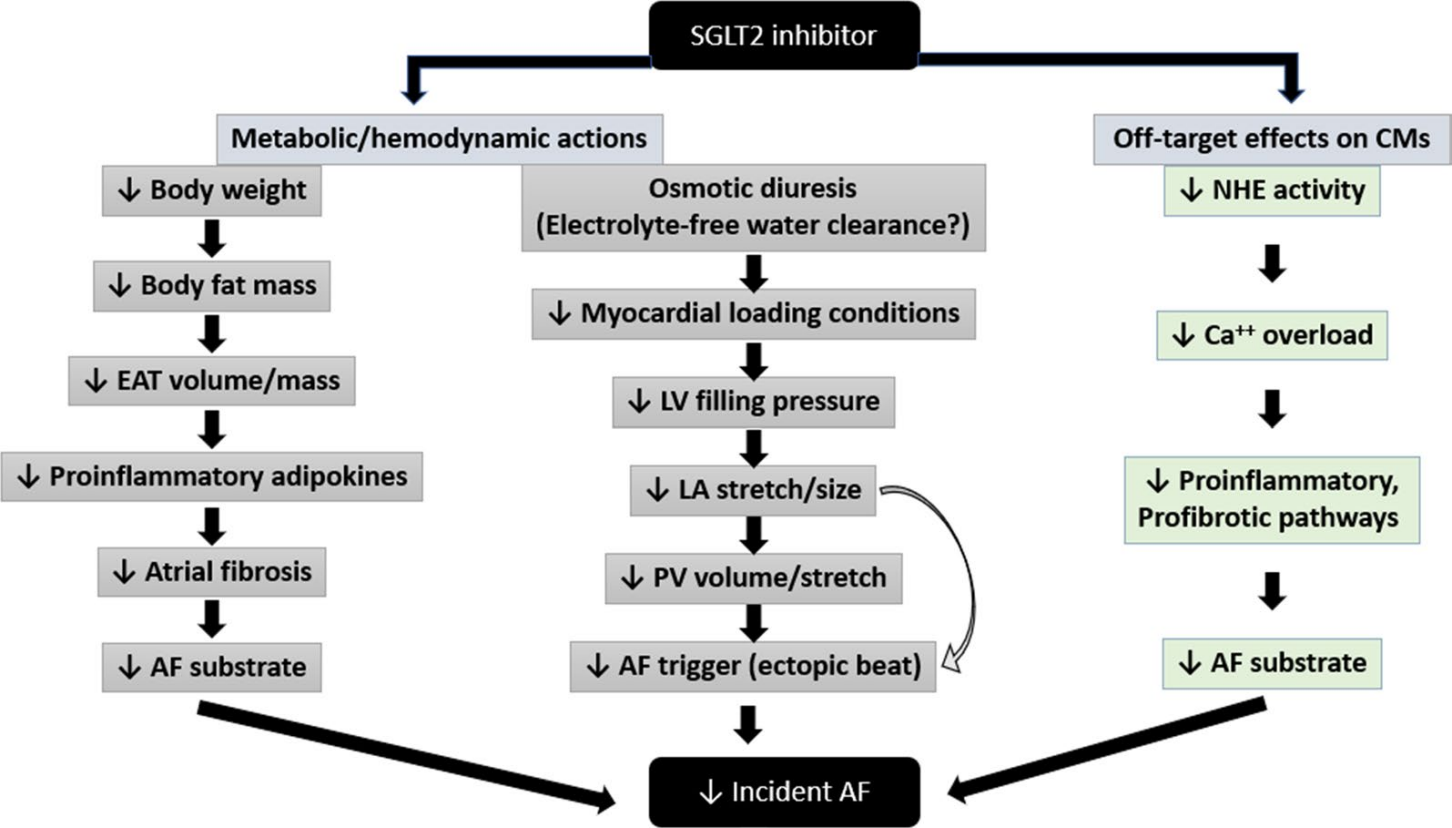
Schematic representation of possible mechanisms by which SGLT2 inhibitors reduce incident atrial fibrillation. Abbreviations: epicardial adipose tissue (EAT); sodium-hydrogen exchanger (NHE); left ventricular (LV); left atrial (LA); pulmonary vein (PV); cardiomyocytes (CM)

Stretch of the pulmonary veins triggers arrhythmia as discussed earlier. Elevated left atrial pressure resulting from left ventricular diastolic dysfunction is transmitted to the pulmonary veins given the anatomical continuity between the two. This leads to increased pulmonary vein volume and dilated vein orifices [109] and is predictive of AF [144]. It is thus important to investigate if amelioration of diastolic dysfunction by SGLT2 inhibitors has a direct upstream effect on pulmonary vein dimensions using computed tomography (CT) in longitudinal studies. A visual representation of potential mechanisms mediating reduction in AF by SGLT2 inhibitors is shown in Fig. 1.

Mechanistic studies with SGLT2 inhibitors may be supported by analysis of circulating biomarkers or fibrosis including type I procollagen C-terminal propeptide (a marker of collagen type 1 synthesis), type III collagen N-terminal propeptide (a marker of collagen type 3 synthesis), [145, 146] and on inflammation including high-sensitivity C-reactive protein, interleukin-6, and tumor necrosis factor alpha [147]. Thus, an expanded analysis involving functional, structural, and biochemical assessments will likely yield valuable clues regarding the clinical significance of the potential beneficial effects of SGLT2 inhibitors and their biological bases.

## Conclusions

The post hoc analysis of the DECLARE-TIMI 58 trial demonstrated a reduction in AF incidence in diabetes mellitus patients treated with SGLT2 inhibitors. The mechanism for this reduction in AF is unknown. Possible mechanisms for the SGLT2 inhibitor-induced reduction in incident AF include reduction in epicardial fat inhibiting pathological atrial remodeling and rapid improvement in left ventricular diastolic function ameliorating hemodynamic stress on the atrium and pulmonary veins. Off-target interactions by the drugs potentially mitigating cardiomyocyte ionic imbalance and ameliorating oxidative stress-induced mitochondrial dysfunction are ancillary mechanisms worth exploring as well. Given the modest reductions in hemoglobin A1c and blood pressure in patients treated with SGLT2 inhibitors, these are unlikely to be predominant mechanisms by which SGLT2 inhibitors reduce AF. Biochemical, functional, and structural studies as highlighted in this review will further help elucidate the mechanistic bases for the observed beneficial effects of SGLT2 inhibitors on incident AF.

## Data Availability

Not applicable.

## Abbreviations

AF: Atrial fibrillation
AFL: Atrial flutter
SGLT2: Sodium-glucose cotransporter 2
EAT: Epicardial adipose tissue
E/e’: Early mitral inflow velocity relative to early diastolic left ventricular relaxation
